# Peripoperative Mortalität nach ICD-Implantation

**DOI:** 10.1007/s00059-021-05033-2

**Published:** 2021-04-16

**Authors:** Harilaos Bogossian, Dimitrios Panteloglou, Zana Karosiene, Susanne Macher-Heidrich, Heinz Jürgen Adomeit, Bernd Lemke, Carsten W. Israel

**Affiliations:** 1 Klinik für Kardiologie und Rhythmologie, Ev. Krankenhaus Hagen, Brusebrinkstr. 20, 58135 Hagen, Deutschland; 2 Universität Witten/Herdecke, Witten, Deutschland; 3 Klinik für Kardiologie, Elektrophysiologie und Angiologie, Klinikum Lüdenscheid, Lüdenscheid, Deutschland; 4 Ärztekammer Nordrhein, Düsseldorf, Deutschland; 5 Ärztekammer Westfalen-Lippe, Münster, Deutschland; 6 Klinik für Kardiologie, Evangelisches Klinikum Bethel, Bielefeld, Deutschland

**Keywords:** Defibrillatoren, Operation, Komplikationen, Risikofaktoren, Qualitätssicherung, Defibrillators, Surgery, Complications, Risk factors, Quality assurance

## Abstract

**Hintergrund:**

Implantierbare Kardioverter-Defibrillatoren (ICD) sind zum Schutz vor plötzlichem Herztod bei Patienten mit primär- oder sekundärprophylaktischer Indikation etabliert. Wie bei allen komplexen operativen Verfahren verbleibt auch bei der ICD-Implantation ein Risiko für Komplikationen bis hin zum Tod. Gegenstand der vorliegenden Arbeit ist es, anhand der Datensätze zur obligaten Qualitätssicherung in Nordrhein-Westfalen die prozedurbezogene Mortalität nach ICD-Implantation zu analysieren.

**Methoden:**

Aus den Datensätzen erfolgte eine Analyse der stationären Todesfälle bei allen 18.625 ICD-Implantationen der Jahre 2010 bis 2012.

**Ergebnisse:**

Während des stationären Aufenthalts verstarben 118 Patienten (0,6 %) nach ICD-Implantation. Patienten im Alter über 80 Jahre (7 %) zeigten eine höhere Mortalität (1,9 % vs. 0,5 % bei < 80-jährigen Patienten, *p* > 0,001), ebenso Frauen (0,95 % vs. 0,54 % bei Männern; *p* = 0,004) und Patienten mit hoher NYHA(New York Heart Association)-Klasse (0,3 % bei NYHA II, 0,7 % bei NYHA III, 3,4 % bei NYHA IV; *p* < 0,001 für alle Vergleiche). Das Vorliegen von Diabetes mellitus (23 % des Kollektivs) beeinflusste die perioperative Letalität nicht, während eine dialysepflichtige Niereninsuffizienz eine signifikant erhöhte Mortalität aufwies (*p* < 0,001 gegenüber Patienten mit Kreatinin ≤ 1,5 mg/dl; *p* = 0,002 gegenüber nicht dialysepflichtigen Patienten mit Kreatinin > 1,5 mg/dl). Patienten mit sekundärprophylaktischer ICD-Indikation wiesen eine signifikant höhere Mortalität auf (1,2 % vs. 0,4 %; *p* < 0,001), die sich beim Auftreten von Komplikationen von 0,6 % auf 3,7 % erhöhte (*p* < 0,001).

**Schlussfolgerung:**

Die operationsbezogene Mortalität bei ICD-Implantation ist bei Patienten über 80 Jahre, hoher NYHA-Klasse, Dialysepflicht, sekundärprophylaktischer Indikation und nach Auftreten von Komplikationen erhöht.

## Abstract (en)

**Background:**

Implantable cardioverter defibrillators (ICD) represent an established treatment in preventing sudden cardiac death in patients with indications for primary or secondary prophylaxis. As for all complex surgical procedures there remains a risk for the occurrence of complications including death also for ICD implantation. The aim of the present study was to analyze the procedure-related mortality in patients after ICD implantation using the data from the obligatory quality assurance program in North-Rhine/Westphalia.

**Methods:**

Data of all 18,625 patients from the quality assurance datasets who underwent ICD implantation in the years 2010–2012 were analyzed.

**Results:**

During the in-hospital stay 118 patients (0.6%) died after ICD implantation. Patients > 80 years old had a higher mortality (1.9% vs. 0.5% in patients < 80 years old, *p* < 0.001) as well as women (0.95% vs. 0.54% in men, *p* = 0.004) and patients with higher New York Heart Association (NYHA) class (0.3% for NYHA II, 0.7% for NYHA III, 3.4% for NYHA IV, *p* < 0.001 for all comparisons). The presence of diabetes mellitus (23% of the collective) did not influence the perioperative mortality, whereas renal failure requiring dialysis showed a significantly increased mortality (*p* < 0.001 compared to patients with creatinine ≤ 1.5 mg/dl and *p* = 0.002 for patients with creatinine > 1.5 mg/dl not requiring dialysis). Patients with indications for ICD secondary prophylaxis had a significantly higher mortality (1.2% vs. 0.4%, *p* < 0.001), which increased from 0.6% to 3.7% (*p* < 0.001) with the occurrence of complications.

**Conclusion:**

The procedure-related mortality after ICD implantation is increased in patients over 80 years of age, higher NYHA class, patients requiring dialysis, in secondary prevention indications and after the occurrence of perioperative complications.

## Einleitung

Die Einpflanzung eines implantierbaren Kardioverter-Defibrillators (ICD) ist ein etabliertes Verfahren zum Schutz von Patienten mit hohem Risiko für das Auftreten des plötzlichen Herztodes [1, 2].

Gegenüber der Herzschrittmacherimplantation ist die Inzidenz der perioperativen Letalität bei ICD-Implantationen niedriger [3]. In den deutschen Registerdaten wird die In-hospital-Mortalität zwischen 0,1 % und 1 % beschrieben [4].

In Nordrhein-Westfalen (NRW) fanden in den Jahren von 2010 bis 2012 18.625 stationäre ICD-Implantationen statt. Für alle diese stationären Implantationen ist eine Qualitätssicherung (QS) mit Angabe von Basisdaten, Operationsdaten inklusive Komplikationen und Outcome-Daten (z. B. Patient nach Hause entlassen, verlegt oder verstorben) verpflichtend. Da die Anzahl an ambulanten ICD-Implantationen verschwindend gering ist, bilden diese Datensätze der QS NRW praktisch alle konsekutiven ICD-Implantationen dieser 3 Jahre ab. Das Versterben während des stationären Aufenthalts zur ICD-Implantation stellt dabei ein Sentinelereignis dar, das in jedem Fall von der betroffenen Klinik erläutert werden muss.

In der vorliegenden Arbeit sollen aus den Datensätzen der QS NRW die Baseline- und Implantationsparameter identifiziert werden, die mit einer erhöhten perioperativen Mortalität nach ICD-Implantation assoziiert sind.

## Methode

In der aktuellen Studie wurden die Datensätze der ICD-Implantationen in NRW von 2010 bis 2012 ausgewertet. Für die Auswertung wurde das Datenmaterial verwendet, das der Geschäftsstelle der QS NRW im Rahmen der gesetzlichen stationären externen Qualitätssicherung vorliegt (insgesamt 18.625 Implantationen: 8507 Einkammer-ICD, 4364 Zweikammer-ICD, 5596 kardiale Resynchronisationstherapie [CRT], 158 sonstige ICD). Aus dem Datenmaterial wurden folgende Parameter über den stationären Aufenthalt der Patienten analysiert:

- Patientencharakteristika: Alter, Geschlecht, NYHA(New York Heart Association)-Klasse, ASA(American Society of Anesthesiologists)-Klassifikation [5], Diabetes mellitus (vorhanden/nicht vorhanden/insulinpflichtig), Niereninsuffizienz (Kreatinin: < 1,5/1,5–2,5/> 2,5 mg/dl ohne und mit Dialyse), Vorliegen einer koronaren Herzkrankheit (KHK) oder Kardiomyopathie;
- Echokardiographie (linksventrikuläre Ejektionsfraktion, LVEF);
- Indikation zur ICD-Implantation;
- perioperative Komplikationen während des stationären Aufenthalts (Reanimation, Pneumothorax, Hämatothorax, Perikarderguss, Taschenhämatom, Sondendislokation, Wundinfektion, sonstige);
- Art der Entlassung (nach Hause, Verlegung in anderes Krankenhaus, Rehabilitation oder Heim, Patient verstorben).

Im „strukturierten Dialog“, werden im Rahmen des Indikators „Mortalität“ verbindlich Fragebögen ausgefüllt, die sich auf verstorbene Patienten beziehen.

Mit Einschätzung des Operateurs findet eine Beurteilung darüber statt, ob aufgetretene Komplikationen ursächlich oder mitursächlich für den Tod des Patienten verantwortlich waren. Die Komplikationsart und der zeitliche Zusammenhang zur Implantation müssen angegeben werden. Sollte weder eine Komplikation noch eine Herzrhythmusstörung für den Tod des Patienten verantwortlich sein, muss dies ausdrücklich vermerkt werden. Ein unsicherer Zusammenhang zwischen dem Tod des Patienten und einer Komplikation der ICD-Implantation wird ebenfalls dokumentiert. Zudem macht der Operateur Angaben zu Begleiterkrankungen, die im Rahmen der ICD-Implantation hinsichtlich der Prognose sowie des peri- und postoperativen Verlaufs einen beeinflussenden Charakter besitzen. Die Angaben erheben keinen Anspruch auf Vollständigkeit. Der Operateur muss dokumentieren, ob nach der ICD-Implantation ein Röntgenthorax, eine Echokardiographie und eine ICD-Kontrolle stattgefunden haben, und dabei den zeitlichen Zusammenhang mit der Implantation berücksichtigen. Für die Analyse des „strukturierten Dialogs“ liegen nur die Daten für die Jahre von 2010 bis 2011 vor.

### Statistische Analyse

Es erfolgte eine deskriptive Analyse des Datenmaterials. Die statistische Signifikanz der Ergebnisse wurde mittels Chi-Quadrat-Tests nach Pearson berechnet (Signifikanzniveau: *p* ≤ 0,05). Die statistische Auswertung der Ergebnisse erfolgte unter Verwendung der Statistik- und Analysesoftware SPSS. Die Darstellung erfolgt in tabellarischer Form sowie in Form von Balkendiagrammen.

## Ergebnisse

### Patientenkollektiv und Altersverteilung

In den Jahren von 2010 bis 2012 erhielten 18.625 Patienten einen ICD. Während des stationären Aufenthalts verstarben 118 Patienten (0,6 %). Beim weiblichen Geschlecht zeigt sich eine signifikant höhere Mortalität (männlich: 79 von 14.508 [0,54 %]; weiblich: 39 von 4117 [0,95 %; *p* = 0,004]).

Das Alter der Patienten betrug zum Zeitpunkt der ICD-Implantation im Mittel 66 Jahre (Median: 69; Minimum: < 1, Maximum: 91 Jahre), das der Verstorbenen im Mittel 71 Jahre (Median: 74; Minimum: 25, Maximum: 88 Jahre). Die Patienten wurden in die folgenden Altersgruppen eingeteilt:

- ≤ 60 Jahre (*n* = 5212; 27,98 %),
- 61–80 Jahre (*n* = 12.020; 64,54 %),
- > 80 Jahre (*n* = 1393; 7,48 %).

**Figure 1:**
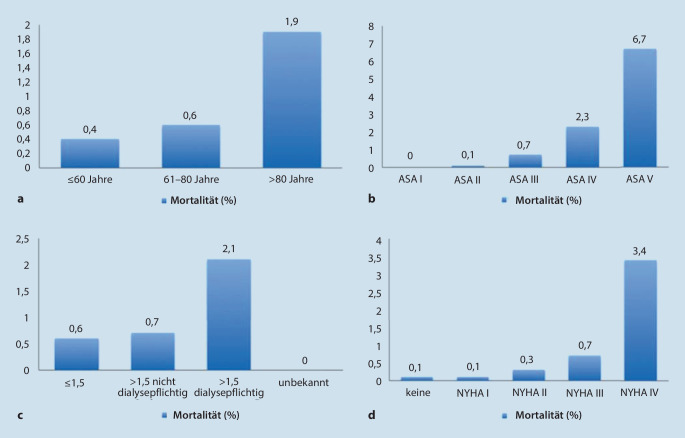

Die Überlebenswahrscheinlichkeit zwischen den ersten beiden Altersgruppen zeigte keinen signifikanten Unterschied (*p* = 0,0748). Die Patienten der dritten Altersgruppe hatten gegenüber den beiden jüngeren Gruppen eine signifikant höhere Mortalität (jeweils* p* < 0,001; Abb. 1; Tab. 1).

**Table 1:** 

	Gesamt	Gesamt %	Tote	Tote %
*Alter*				
≤ 60 Jahre	5212	27,98	20	0,4
61–80 Jahre	12.020	64,54	72	0,6
> 80 Jahre	1393	7,48	26	1,9
≤ 60 Jahre und > 80 Jahre: *p* < 0,001 61–80 Jahre und > 80 Jahre: *p* < 0,001 ≤ 60 Jahre und 61–80 Jahre: *p* = 0,0748				
*Geschlecht*				
Männlich	14.508	77,9	79	0,5
Weiblich	4117	22,1	39	1
Männlich vs. Weiblich: *p* = 0,004				
*ASA-Klassifikation*				
ASA I	429	2,3	0	0
ASA II	4817	25,9	6	0,1
ASA III	12.268	65,9	86	0,7
ASA IV	1096	5,9	25	2,3
ASA V	15	0,1	1	6,7
Wegen geringer Fallzahl wird für die ASA-Klassen I und V keine Signifikanz angegeben. ASA II vs. III: *p* < 0,001 ASA II vs. IV: *p* < 0,001 ASA III vs. IV: *p* < 0,001				
*NYHA-Klasse*				
Keine	990	5,3	1	0,1
NYHA I	1148	6,2	1	0,1
NYHA II	6229	33,4	21	0,3
NYHA III	9517	51,1	70	0,7
NYHA IV	741	4	25	3,4
NYHA II vs. NYHA III: *p* < 0,001 NYHA II vs. NYHA IV: *p* < 0,001 NYHA III vs. NYHA IV: *p* < 0,001				
*Ejektionsfraktion (EF)*				
EF unbekannt	343	1,8	5	1,5
EF > 50 %	1328	7,1	6	0,5
EF 50 bis > 35 %	1981	10,6	11	0,6
EF ≤ 35 %	14.973	80,4	96	0,6
EF ≤ 35 % und 35–50 %: *p* = 0,6506 EF ≤ 35 % und > 50 %: *p* = 0,4				

*ASA* American Society of Anesthesiologists, *NYHA* New York Heart Association

### ASA-Klassifikation, NYHA-Stadium und linksventrikuläre Ejektionsfraktion

Die Mortalität war bei Patienten mit höherer ASA-Klasse gegenüber Patienten mit niedriger ASA-Klasse signifikant höher. Wegen geringer Fallzahl in den ASA-Klassen I und V konnte keine adäquate Signifikanzberechnung erfolgen. Die Ermittlung der Signifikanzen zwischen den Gruppen ASA II–IV ergab folgende Werte (Abb. 1; Tab. 1):

- ASA II zu III: *p* < 0,001;
- ASA II zu IV: *p* < 0,001;
- ASA III zu IV: *p* < 0,001.

Die Mortalität bei Patienten höherer NYHA-Klassen war signifikant höher als bei Patienten niedriger NYHA-Klassen. Bei NYHA I erfolgte wegen der geringeren Fallzahl keine Signifikanzberechnung. Die übrigen Signifikanzen wurden wie folgt berechnet (Abb. 1; Tab. 1):

- NYHA II und NYHA III: *p* < 0,001;
- NYHA II und NYHA IV: *p* < 0,001;
- NYHA III und NYHA IV: *p* < 0,001.

Bei der Unterteilung der Patienten nach ihrer EF wurden 4 Gruppen gebildet (EF > 50 %, EF > 35–50, EF ≤ 35, EF unbekannt; Tab. 1). Entsprechend der ICD-Indikationen war die Gruppe von Patienten mit einer EF von 35 % oder weniger am größten (> 80 % der Patienten). Die Mortalität zeigte keine signifikanten Unterschiede zwischen den hier gebildeten Gruppen (Tab. 1):

- EF ≤ 35 % vs. > 35–50 %: *p* = 0,6506;
- EF ≤ 35 % vs. > 50 %: *p* = 0,4.

### Komorbidität, Kardiomyopathien, führende Indikation und Komplikationen

Die Analyse der perioperativen Mortalität der Patienten mit und ohne Diabetes mellitus zeigte keine signifikanten Unterschiede (Tab. 1):

- kein Diabetes mellitus vs. nicht insulinpflichtiger Diabetes mellitus: *p* = 0,452;
- kein Diabetes mellitus vs. insulinpflichtiger Diabetes mellitus: *p* = 0,892;
- kein Diabetes mellitus vs. Diabetes mellitus (insulinpflichtig und nicht insulinpflichtig): *p* = 0,61.

Dagegen war die dialysepflichtige Niereninsuffizienz sowohl gegenüber Patienten ohne Niereninsuffizienz als auch gegenüber nicht dialysepflichtigen niereninsuffizienten Patienten mit einer signifikant höheren Mortalität assoziiert (Tab. 1; Abb. 1).

Die Verteilung der Kardiomyopathien ist in Tab. 3 dargestellt. Bei den beiden größten Gruppen (ischämische [ICM] und dilatative Kardiomyopathie [DCM]) zeigten sich keine signifikanten Unterschiede hinsichtlich der Mortalität (*p* = 0,228). Auch die Analyse der KHK im Speziellen zeigte hinsichtlich der perioperativen Mortalität keine signifikanten Unterschiede. Die Mortalität bei Patienten mit und ohne KHK war gleich (Tab. 2):

- KHK mit Myokardinfarkt in der Anamnese vs. Patienten ohne KHK: *p* = 0,581;
- KHK mit Myokardinfarkt in der Anamnese vs. KHK ohne Myokardinfarkt in der Anamnese: *p* = 0,375.

**Table 2:** 

	Gesamt	Gesamt %	Tote	Tote %
Diabetes mellitus (DM)				
Kein DM	14.259	76,6	88	0,6
Nicht insulinpflichtiger DM	2837	15,2	21	0,7
Insulinpflichtiger DM	1529	8,2	9	0,6
Kein Diabetes mellitus vs. Diabetes mellitus (nicht insulinpflichtig): *p* = 0,452 Kein Diabetes mellitus vs. Diabetes mellitus (insulinpflichtig): *p* = 0,892 Kein Diabetes mellitus vs. Diabetes mellitus (insulinpflichtig und nicht insulinpflichtig): *p* = 0,61				
*Niereninsuffizienz*				
Kreatinin ≤ 1,5 mg/dl	14.856	79,8	88	0,6
Kreatinin > 1,5 mg/dl (nicht dialysepflichtig)	3177	17,1	21	0,7
Kreatinin > 1,5 mg/dl (dialysepflichtig)	423	2,3	9	2,1
Unbekannt	169	0,9	0	0
Kreatinin ≤ 1,5 mg/dl vs. > 1,5 mg/dl (dialysepflichtig): *p* < 0,001 Kreatinin ≤ 1,5 mg/dl vs. > 1,5 mg/dl (nicht dialysepflichtig): *p* = 0,65 Kreatinin > 1,5 mg/dl (nicht dialysepflichtig) vs. > 1,5 mg/dl (dialysepflichtig): *p* = 0,002				
*Koronare Herzkrankheit (KHK)*				
KHK ohne MI in der Anamnese	4473	24	25	0,6
KHK mit MI in der Anamnese	7357	39,5	51	0,7
Nein	6795	36,5	42	0,6
KHK mit MI vs. keine KHK: *p* = 0,581 KHK mit MI vs. KHK ohne MI: *p* = 0,375				

*MI* Myokardinfarkt

**Table 3:** 

Kardiale Grunderkrankung	Gesamt	Gesamt %	Tote	Tote %
Keine	481	2,6	0	0
ICM	10.506	56,4	73	0,7
DCM	5939	31,9	32	0,5
Hypertensive Herzkrankheit	329	1,8	1	0,3
Erworbene Herzklappenfehler	172	0,9	3	1,7
Angeborene Herzklappenfehler	60	0,3	0	0
Brugada-Syndrom	91	0,5	0	0
Kurzes QT-Syndrom	0	0	0	0
Langes QT-Syndrom	144	0,8	0	0
HCM	397	2,1	2	0,5
ARVC	48	0,3	0	0
Sonstige	458	2,5	7	1,5
ICM vs. DCM: *p* = 0,228				

*ICM* ischämische Kardiomyopathie, *DCM* dilatative Kardiomyopathie, *HCM* hypertrophe Kardiomyopathie, *ARVC* arrhythmogene rechtsventrikuläre Kardiomyopathie

**Table 4:** 

Führende Indikation	Gesamt	Gesamt %	Tote	Tote %
Primärpräventiv	13.255	71,2	55	0,4
Sekundärpräventiv	5370	28,8	63	1,2
Primärpräventiv vs. sekundärpräventiv: *p* ≤ 0,001				
*Komplikationen*				
Nein	18.271	98,1	105	0,6
Ja	354	1,9	13	3,7
Auftreten von Komplikationen vs. keine Komplikationen: *p* < 0,001				

**Figure 2:**
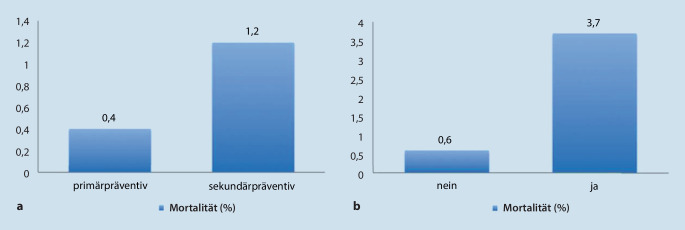

Hinsichtlich der Implantationsindikation zeigte sich die Mortalität bei Patienten mit primärpräventiver ICD-Indikation gegenüber der sekundärpräventiven Indikation niedriger (*p* ≤ 0,001; Abb. 2a).

Bei 1,9 % der Patienten traten peri- oder postoperative Komplikationen (kardiopulmonale Reanimation, Pneumothorax, Hämatothorax, Perikarderguss, Taschenhämatom, Sondendislokation, Wundinfektion oder sonstige) auf; bei diesen Patienten lag die Mortalität mit 3,7 % signifikant höher als bei Patienten ohne perioperative Komplikationen (0,6 %; *p* < 0,001; Abb. 2b).

### Prozedurbedingte Mortalität

Für die Jahre 2010 und 2011 liegen die Fragebögen des „strukturierten Dialogs“ vor. Demnach wurden die Fälle der 89 verstorbenen Patienten (von insgesamt 12.121 ICD-versorgten Patienten in den Jahren 2010–2011) im Einzelnen mit den implantierenden Kliniken/Operateuren diskutiert. Daraus resultierte, dass nur 4 Patienten (0,033 %) unmittelbar perioperativ verstorben sind.

## Diskussion

Die vorliegende Analyse von fast 20.000 ICD-Implantationen zeigt, dass eine ICD-Implantation bei folgenden Patienten bzw. Bedingungen mit einer erhöhten Mortalität noch während des stationären Aufenthalts nach Implantation assoziiert ist:

- Alter > 80 Jahre,
- weibliches Geschlecht,
- schlechterer Allgemeinzustand bzw. fortgeschrittene Herzinsuffizienzsymptomatik (ASA-Klasse, NYHA-Klasse),
- terminale Niereninsuffizienz mit Notwendigkeit einer Dialyse,
- sekundärprophylaktische ICD-Indikation (Tab. 4),
- Auftreten von Komplikationen während oder nach der Implantation (Tab. 4).

Die Beurteilung von QS-Daten wird kontrovers diskutiert [6]. Die vorgestellten Daten sind jedoch aufgrund ihrer praktisch lückenlosen Abbildung der Mortalität bei einer so großen Zahl konsekutiver, nichtselektionierter ICD-Implantationen von beträchtlicher klinischer Relevanz. Nicht zuletzt weil „Tod“ einen „harten“ Endpunkt darstellt, der aufgrund der Verknüpfung der Krankenhaussoftware überprüfbar ist und kaum fehldokumentiert werden kann, ist die Validität der Daten als sehr hoch einzustufen.

Die Ergebnisse können für operierende Ärzte von hoher Relevanz sein, da sie helfen, Patienten zu identifizieren, die ein besonderes Risiko haben, noch während des stationären Aufenthalts zu versterben und von der ICD-Implantation keinen Nutzen zu erlangen. Die Ergebnisse können insbesondere für die Patientenaufklärung hilfreich sein, das individuelle perioperative Risiko genauer einzustufen.

Die Indikationen zur ICD-Implantation sind sowohl zur Primär- als auch zur Sekundärprävention etabliert [2, 7–10], obwohl sie auf älteren Studien basieren. Es liegen viele Langzeitdaten zu Komplikationen und Mortalität vor [11–13]. Hinsichtlich der perioperativen Mortalität basieren die Daten insbesondere auf Registerdaten [4, 14, 15]. Die vorliegende Auswertung der Datensätze der QS NRW ist vergleichbar mit ausländischen Ergebnissen, denen zufolge die Mortalität bei der ICD-Implantation tatsächlich niedriger liegt als bei Schrittmacherimplantationen [3]. Über die Ursachen dessen kann nur spekuliert werden (jüngeres Alter der Patienten mit ICD- im Vergleich zur Schrittmacherimplantation, Operation in erfahreneren Institutionen bzw. von erfahreneren Implanteuren, geringere Komorbidität?). Im Unterschied zur Schrittmacherimplantation fordert die aktuelle QS für Patienten mit ICD-Implantation eine mindestens 1‑jährige Lebenserwartung in gutem funktionellen Status.

Das geschlechtsspezifische Outcome variiert in der Literatur stark. Neben Studien, in denen kaum relevante geschlechtsspezifische Unterschiede beobachtet wurden [16], konnte in anderen gezeigt werden, dass Komplikationen bei Frauen häufiger auftreten [17]. Hierbei sind insbesondere der Pneumothorax und die Perikardtamponade zu nennen [18]. In der vorliegenden Auswertung zeigte sich eine perioperativ signifikant erhöhte Mortalität bei Frauen. Kanadische Kardiologen haben die ICD-Versorgung von Männern und Frauen analysiert und berichtet, dass – trotz Adjustierung – Männer nach Myokardinfarkt oder mit Herzinsuffizienz 3‑mal häufiger mit einem ICD versorgt werden [19]. Dies lässt vermuten, dass Frauen nur bei schwerer Herzinsuffizienz versorgt werden und somit eine höhere Anfälligkeit haben könnten. Eine höhere Rate an Komplikationen bei Frauen (Blutungen, Wundheilungsstörungen etc.) wurde in vielen Studien mit invasiven bzw. operativen Therapien nachgewiesen.

Al-Khatib et al. haben wesentliche Risikofaktoren der Sterblichkeit definiert. Hierunter zählen unter anderem [17]:

- Herzinfarkt in der Anamnese,
- Diabetes mellitus,
- Niereninsuffizienz und Herzinsuffizienz.

Hinsichtlich der perioperativen Mortalität haben sich in den aktuell ausgewerteten Daten nur die dialysepflichtige Niereninsuffizienz und die Herzinsuffizienz, basierend auf der NYHA-Klasse, aber nicht die EF als signifikante Marker bestätigt. Trotzdem sollte Dialysepatienten eine ICD-Implantation nicht vorenthalten werden, da auch neue Daten zeigen, dass Dialysepatienten von einer sekundärprophylaktischen ICD-Implantation profitieren [20].

Die vorliegenden Daten zeigen ein 3‑fach erhöhtes Risiko bei der sekundärprophylaktischen ICD-Implantation. Dies ist am ehesten auf dem Boden der akuten Arrhythmielast mit möglicherweise auch reduziertem Status der Patienten vor der Operation (z. B. nach Reanimation) zu erklären. Daher sollte der Zeitpunkt der ICD-Implantation bei diesen Patienten kritisch gewählt werden. Das Hauptargument gegen ein längeres Zuwarten ist die Gefahr eines Arrhythmierezidivs. Allerdings sollte in einem stabilen Zustand und nicht unmittelbar nach einem VT(ventrikuläre Tachykardie)-Sturm operiert werden. Dabei gilt es abzuwägen, ob im stationären Setting eine protrahierte Implantation erfolgen oder sogar nach Versorgung mit tragbarer Defibrillatorweste der Zeitpunkt der Operation weiter verschoben werden sollte. Auch eine frühzeitige VT-Ablation vor der ICD-Implantation ist Gegenstand aktueller Studien [21].

Unsere Daten sollten dazu genutzt werden, bei multimorbiden Patienten bereits im Vorfeld an ein erhöhtes Risiko für Komplikationen und perioperative Mortalität zu denken und daher bei jedem Schritt der Implantation besondere Sorgfalt walten zu lassen. Dies bekräftigen auch die Daten aus dem „strukturierten Dialog“. Einerseits ist das sehr niedrige operationsassoziierte Risiko als positiv zu bewerten, und es bestätigt die sichere Durchführung der Implantationen, andererseits rückt der Appell für die korrekte Auswahl der Patienten weiter in den Vordergrund, da die Mortalität überwiegend durch die Komorbidität der Patienten während des stationären Aufenthalts erklärt ist.

Bei der Implantation von Ein- und Zweikammer-ICD ist eine Komplikationsrate von 1,0–4,5 % beschrieben [4]. In der aktuellen Auswertung bei 18.625 Patienten betrug die Inzidenz von Komplikationen 1,9 %. Diese Patienten bedürfen postoperativ einer besonderen Aufmerksamkeit, da ein signifikant (rechnerisch 6‑fach) erhöhtes Risiko einer perioperativen Mortalität besteht (3,7 % vs. 0,6 %; *p* < 0,001).

Das perioperative Mortalitätsrisiko ist in der vorliegenden Analyse bei Patienten mit sekundärprophylaktischer Indikation mit 1,2 % signifikant höher als bei solchen mit primärprophylaktischer Indikation (0,4 %; *p* ≤ 0,001). Dies beschreibt allerdings nur die akute Beobachtung. Hinsichtlich des Langzeitbeobachtung liegen in der Literatur auch entgegengesetzte Daten vor. So besteht bei Patienten nach primärprophylaktischer Implantation (nach der ersten ICD-Auslösung) eine höhere Arrhythmielast als bei Patienten nach sekundärprophylaktischer Implantation [22].

Zusammengefasst präsentieren die analysierten Daten einer großen Patientenkohorte, dass es, obwohl die perioperative Mortalität bei der ICD-Implantation mit 0,6 % niedrig ist, Patienten mit deutlich erhöhtem Risiko gibt, die identifiziert und entsprechend protektiv behandelt werden sollten. Aufgrund von sowohl neuen kritischen Daten bezüglich der ICD-Therapie [23, 24], verbesserter medikamentöser Herzinsuffizienztherapie, verbesserter Interventions- und Ablationstherapie als auch der Option, längere zeitliche „Risikofenster“ mit tragbaren Defibrillatoren zu überbrücken, sollten insbesondere bei Risikopatienten alle Möglichkeiten genutzt werden, um eine ICD-Implantation, sofern dies sinnvoll sein kann, zu vermeiden.

## Limitationen

Die Ergebnisse liefern wichtige Erkenntnisse über postoperative Komplikationen und das Risikomanagement von ICD-Patienten, die jedoch auf die durch die QS erfassten Daten limitiert sind. Die Analyse ist auf die Krankenhausmortalität beschränkt, und es kann keine Aussage über die Mortalität nach Entlassung oder im Follow-up getroffen werden. Dafür werden hier jedoch sicher alle Todesfälle zum Entlassungszeitpunkt erfasst. Natürlich können nach Entlassung und darüber hinaus noch Patienten verstorben sein, aber deren Anzahl ist als gering einzuschätzen. Diese Limitation resultiert aus der gewählten Methodik, die durch die QS vorgegeben ist.

## Fazit für die Praxis

- Hinsichtlich der perioperativen Mortalität besteht ein signifikant erhöhtes Risiko an einer ICD(implantierbarer Kardioverter-Defibrillator)-Operation zu versterben, wenn die Patienten älter als 80 Jahre oder weiblich sind, ein fortgeschrittenes NYHA(New York Heart Association)-Stadium oder eine dialysepflichtige Niereninsuffizienz aufweisen.
- Patienten, die einen ICD aufgrund einer sekundärprophylaktischen Indikation erhalten, haben ein höheres perioperatives Mortalitätsrisiko.
- Wie bei der Herzschrittmacherimplantation ist auch bei der ICD-Implantation das Auftreten von Komplikationen ein Risikomarker für eine höhere perioperative Mortalität.
